# Use of an Electronic Medication Administration Record (eMAR) for Surveillance of Medication Omissions: Results of a One Year Study of Antimicrobials in the Inpatient Setting

**DOI:** 10.1371/journal.pone.0122422

**Published:** 2015-04-09

**Authors:** Bruce R. Dalton, Deana M. Sabuda, Lauren C. Bresee, John M. Conly

**Affiliations:** 1 Pharmacy Department, Alberta Health Services, Calgary Zone. Calgary, Alberta, Canada; 2 O’Brien Institute for Public Health, Cumming School of Medicine, University of Calgary, Calgary, Alberta, Canada; 3 Department of Community Health Sciences, Cumming School of Medicine, University of Calgary, Calgary, Alberta, Canada; 4 Department of Medicine, Cumming School of Medicine, University of Calgary, Calgary, Alberta; 5 Department of Microbiology, Immunology and Infectious Diseases, Cumming School of Medicine, University of Calgary, Calgary, Alberta, Canada; 6 Department of Pathology and Laboratory Medicine, Cumming School of Medicine, University of Calgary, Calgary, Alberta, Canada; 7 Synder Institute for Chronic Diseases, Cumming School of Medicine, University of Calgary, Calgary, Alberta, Canada; University of Nebraska Medical Center, UNITED STATES

## Abstract

**Introduction:**

Medication administration omissions (MAO) are usually considered medication errors but not all MAO are clinically relevant. We determined the frequency of clinically relevant MAO of antimicrobial drugs in adult hospitals in Calgary, Alberta, Canada based on electronic medication administration record (eMAR).

**Methods:**

We examined 2011 data from eMAR records on medical wards and developed a reproducible assessment scheme to categorize and determine clinical relevance of MAO. We applied this scheme to records from 2012 in a retrospective cohort study to quantify clinically relevant MAO. Significant predictors of clinically relevant MAO were identified.

**Results:**

A total of 294,718 dose records were assessed of which 10,282 (3.49%) were for doses not administered. Among these 4903 (1.66% of total); 47.68% of MAO were considered clinically relevant. Significant positive predictors of clinically relevant MAO included inhaled (OR 4.90, 95% CI 3.54-6.94) and liquid oral (OR 1.32, 95% CI 1.18-1.47) route of medication compared to solid oral and irregular dose schedules. Evening nursing shift compared to night shift (OR 0.77 95% CI 0.70-0.85) and parenteral (OR 0.50, 95% CI 0.46-0.54) were negative predictors, The commonest reasons for relevant MAO were patient preference, unspecified reason, administration access issues, drug not available or patient condition.

**Conclusion:**

Assessment of MAO by review of computer records provides a greater scope and sample size than directly observed medication administration assessments without “observer” effect. We found that MAO of antimicrobials in inpatients were uncommon but were seen more frequently with orally administered antimicrobials which may have significance to antimicrobial stewardship initiatives.

## Introduction

For unclear reasons antimicrobial agents are commonly involved in medication administration errors and omissions.[1,2,3] Antimicrobial usage is common, used in up to 40% of inpatients,[4] and improper administration of antimicrobials could have an important effect on individual patient and hospital level outcomes. Antimicrobials are given for relatively short courses to patients who may have life-threatening conditions and omitted doses may lead to sub-optimal drug concentrations and the development of antimicrobial resistance[5,6] as well as therapeutic failure.

Most published studies to date examining medication administration have used an observer to record events.[1,2,3] The strength of this method is prospective observation, but it also may introduce measurement bias due to the influence of the observer ‘s presence, that is the ‘Hawthorne effect’, where people modify their behaviour because they know they are being observed.[7] Additionally, observer recorded studies are usually of limited scope because of the time constraints. Retrospective review of patient paper charts and medication administration records are very labour intensive and result in small studies of lower quality or significance. A wide range of dose omission rates have been reported (0.58% to 12.60%),[1–3,8–10] possibly due to sample size variation, study setting or other methodologic differences. Electronic medical administration record (eMAR) systems offer an alternative strategy to study adherence to prescriptions in health care institutions and facilitate efficient review of a large number of drug administration events with no potential for observer effect bias. Identification of factors associated with prescription, medication and nursing practice that relate to clinically relevant MAO may inform relevant healthcare professionals and policy makers of potential strategies of intervention in order to reduce omissions. For instance if omission errors occur at an increased frequency between 2–5 am, efforts could be made to routinely schedule doses at other times.

Given this background, we sought to determine the frequency of antimicrobial medication administration omissions (MAO) on acute medicine wards, to identify factors predictive of antimicrobial MAO, and to describe the reasons given by nursing staff for clinically relevant antimicrobial MAO.

## Methods

We performed a retrospective cohort study of patients admitted to medicine wards in the urban area of Calgary, Alberta, Canada. In 2011–12 there were 3 adult acute care sites serving a metropolitan population of about 1.4 million residents. All 3 hospitals were medical school affiliated teaching centres. Hospital 1 was an 1100 bed tertiary referral centre, whereas hospitals 2 and 3 were large urban acute care hospitals each with capacities of 550–650 beds. All three have general medicine wards and medicine teaching units although of the three, hospital 3 is staffed by fewer medical trainees and generally has a lower acuity patient population. Computerized physician order entry (CPOE) and electronic medication administrations (eMAR) on Sunrise Clinical Manager (SCM v. 5.8, Eclipsys Corp 2010, Atlanta, Ga.) have been used in these sites since 2008. Computers on wheels (COWs) are used by nursing staff when recording doses administered at the bedside.

EMAR records for oral, parenteral and inhaled systemic antimicrobials for calendar year 2011 and 2012 were retrieved from the CPOE department. The records from 2011 were used as a feasibility study to examine the size of analysis which would be possible and to develop the methods of classifying omissions. We did not quantify or report the results of the 2011 analysis because the methods were in development as we went through the data so we did not apply a consistent process to this dataset. The 2012 records were used as the study database applying the methods developed using the 2011 data.

The computerized eMAR system, SCM, is the scheduling and documentation system used for medication administration in Calgary hospital inpatient medicine wards. Cells for each dose in an electronic table of medications are created when an order is entered by the prescriber. When a dose is signed off by a nurse at the time of administration, a check appears in the cell corresponding to the time of the scheduled dose and the status of the dose becomes “performed”. If a dose is not given the cell turns red, signalling a dose is beyond the scheduled time and the status of the dose changes to “overdue.” Doses not given maybe signed off as “not performed” and comments may also be included. If a patient is discharged before the dose is signed off as “not performed”, its status remains “overdue”. Individual scheduled doses may be rescheduled or the medication schedule for the order may be altered to adjust for times when a dose cannot be given. MAO were defined as those identified as “not performed” or “overdue” and then assessed for clinical relevance. MAO doses were considered not clinically relevant when a rescheduled dose was given within 50% of the time before the next scheduled dose and all the intended doses for the specific calendar day had been administered. MAO for orders scheduled for only one dose and discontinued within 2 hours of the order creation were assumed to be “cancelled” and regarded as not clinically relevant. MAO were also considered not clinically relevant if a notation in the eMAR was added by the assigned nurse about the intention of treating team to discontinue or change an order the order was conditional and not meeting conditions at the time in question, there was a duplicate order, the drug concentration was high, the patient was on pass or other explanation judged by the study assessor to render the omission irrelevant. Dose omissions for doses scheduled within 6 hours prior to hospital discharge were considered not relevant for the following reasons:

- we assume discharge plans for continuation of therapy have been made,
- we assume that if discharge is due to death, omission of doses within the last 6 hours of life would be unlikely to have had an impact and resuscitative measures may have precluded antimicrobial administration and recording,
- patients may physically leave the hospital considerably earlier than when they are officially discharged in the admission/discharge/transfer system.

Doses signed off as performed within 6 hours of discharge were still compiled as part of our denominator of the rate of MAO because in the case where doses are administered, the patient had not yet been discharged.

**Fig 1:**
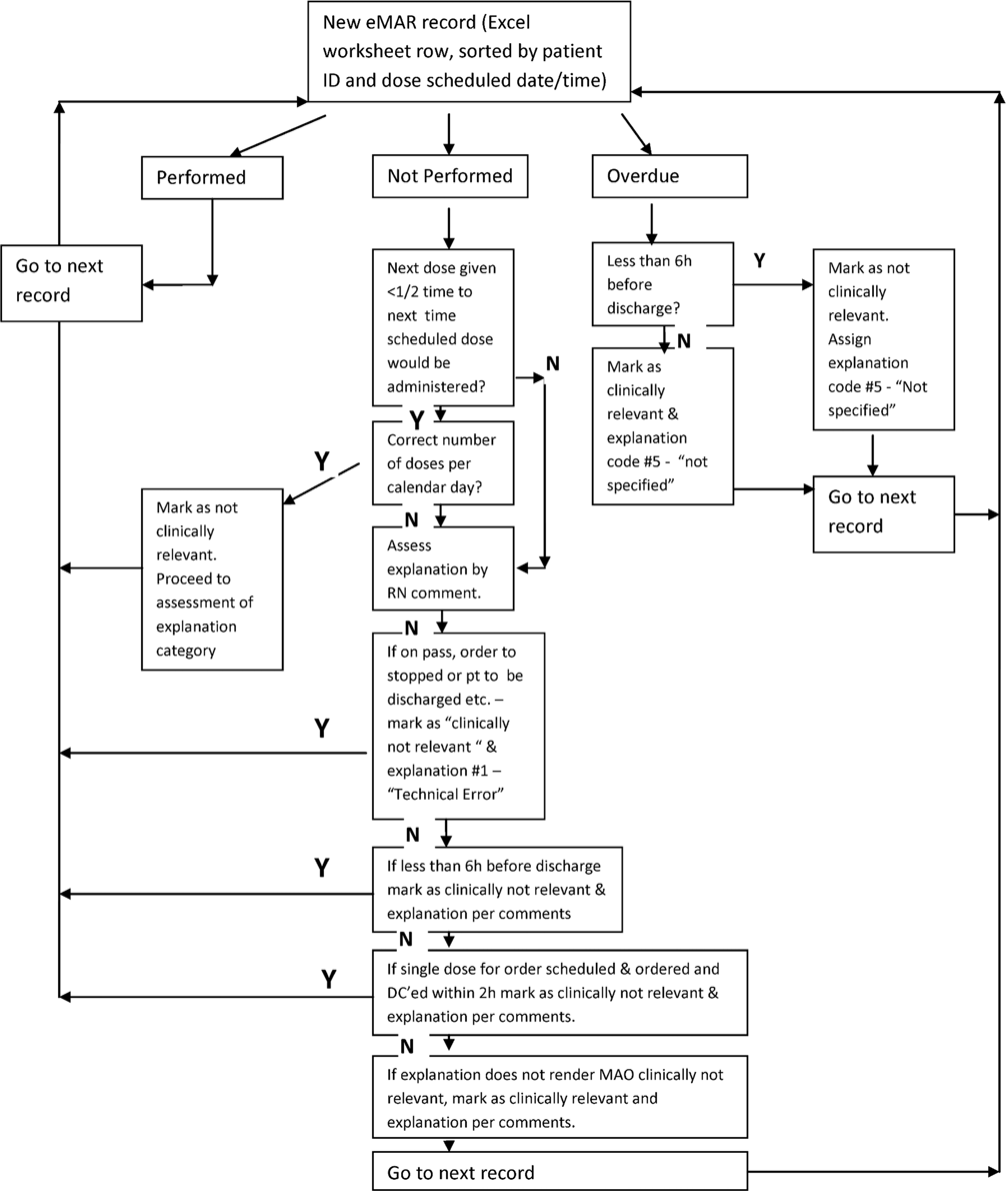
Assessment Process for Electronic Medical Administration Record (eMAR) data.

Aside from classifying MAO by relevance, comments by nurses explaining missed doses were assessed by reason type (ie. “Patient off ward at test”, “no IV site”, “drug not available”, “patient refused” etc.), as outlined in Table 1. Doses documented as “not given” with no explanation were assumed to be missed and clinically relevant, unless discharge from hospital was within 6 hours. Doses missed while patients were on a pass from the hospital were not signed off as an act conducted by the nurses but since passes are arranged with provision made for medications while patients are away from hospital, MAO were documented as not given but not clinically relevant. Fig 1 provides a diagrammatic illustration of the assessment process.

**Table 1:** Classification of Medication Administration Omissions (MAO).

Code/Category	Example of explanation for MAO	Clinically Relevant
1. Technical error	Patient discharged or about to be discharged Order to be discontinued (ie. “Discussed with MD”) Order (to be) held (“discussed with MD”) Duplicate orderPatient on pass Goals of care change Patient deceased	No
2. No intravenous access	IV out and can’t be re-sited Central line placement not yet confirmed Central line malfunction Patient receiving transfusion or other medication	Yes /No (if ‘make up’ dose given)
3. No gastrointestinal access	Patient nauseated/vomiting No NG/OG tube or placement not confirmed NPO	Yes /No (if ‘make up’ dose given)
4. Patient not available	In operating room Patient at procedure	Yes /No (if ‘make up’ dose given)
5. Not specified/other/unclear	No explanation Does not fit other explanations Cannot understand explanation	Yes /No (if ‘make up’ dose given)
6. Drug not available	Not up from pharmacy Precipitate in liquid (quality/contamination issue)	Yes /No (if ‘make up’ dose given)
7. Patient condition	Drowsy or reduced LOC (for oral medication)	Yes /No (if ‘make up’ dose given)
8. Patient preference/refusal	Patient refused/preferred Family refused/preferred Refused line/tube placement	Yes /No (if ‘make up’ dose given)
9. Adverse reaction	Rash, other. Patient believes drug causes adverse effect and refuses	Yes
10. Scheduling issue	Rescheduled First dose ordered and given “stat”, first scheduled dose follows too soon after	No
11. Serum concentration of drug too high	Vancomycin, gentamicin, other.	No
12. Awaiting drug serum concentration	Serum drug concentration drawn but result not yet available	Yes /No (if ‘make up’ dose given)

MD: medical doctor. IV: intravenous. NG: nasogastric tube. OG: orogastric tube. NPO: nothing per os. LOC: level of consciousness.

Data included in the analysis were hospital site, drug name, product type (injection, oral tablet, oral liquid, solution for nebulization), route of administration (intravenous, oral, etc), dose scheduled date/time, dose performed date/time, dose recorded date/time, dose status (performed, overdue, not performed), dose reason (for omission), dose comment, admission date/time, discharge date/time, order schedule (daily, every 4 hours, etc). Nursing shift (day, evening, night) was determined from the date/time scheduled. Rate of omissions were determined by dividing number of omissions by all doses given plus relevant omissions. We excluded non-relevant omissions from the denominator.

Doses scheduled at the following locations at time of administration were excluded: outpatient clinics and emergency wards, operating and recovery rooms and wards housing oncology/bone marrow transplant and renal medicine because documentation using eMAR is usually incomplete and patient time away from these units was considerable.

Quality control. The primary assessor of dose documentation classification reviewed all medication opportunities and categorized them as described above. A convenience sample of ~20,000 patient medication administration records were assessed independently by a second assessor. Cohen’s Kappa was determined for assessment of inter-rater reliability.[11]

### Statistical analysis

Data were retrieved and compiled using Excel (Microsoft, 2007, Redmond, Wa). Cross tabulation of relevant omission rate against independent variables of route of medication, frequency of medication order, nursing shift at time dose scheduled and hospital site was performed with SPSS (v.19, GE Healthcare, Chicago, Ill.) and tested for significance using Chi-squared statistics for categorical variables.[12] Binary multivariate logistic regression was used to determine the adjusted odds ratio and 95% confidence intervals (CI) for associations of clinically relevant omission rate with route, site, ward, shift and number of doses per day using SPSS.[13] A significance level of 0.1 was used for inclusion and 0.05 for exclusion in the multivariate model by enter method. Cohen’s kappa was calculated using cross-tabulation in SPSS.

Approval to conduct this study was granted by the Conjoint Health Research Ethics Board of the University of Calgary and waived need for individual patient consent (ID number; E-25256).

## Results

In three adult hospitals in Calgary in calendar year 2012 there were 10916 patients treated on medical wards with antimicrobial agents in 13383 admissions. A total of 294,718 doses were scheduled and assessed for performance and if omitted, the reason for the omission as well as the clinical relevance was determined. A second independent assessor analyzed 21,536 dose administration records which included 653 unperformed doses. Agreement of reason for omission with a second assessor was good (k = 0.68, p<0.001), as was the assessment of relevance (k = 0.75, p<0.001). A total of 96.51% of antimicrobial doses were given as scheduled. In total 10,282 (3.49%) doses were not given, of which 4903 (1.67%) were considered clinically relevant omissions. Daily, two, three and four times per day dosing frequency accounted for 96.03% of doses given. The relevant omission rate by frequency was between 1–2% for most frequencies except “every 3 days,” “3 times per week” and “5 times per day,” all of which were uncommonly used regimens (Table 2). Clinically relevant MAO rate by route of administration and nursing shift are detailed in Table 2. Solid oral and liquid oral were omitted approximately 2 and 2.5 times more often, respectively, than intravenously administered drugs. The nursing shift associated with the lowest clinically relevant MAO rate was the night shift (1.38 MAO/100 doses), which is also when the fewest number of antimicrobial doses are scheduled (Table 3).

**Table 2:** Bivariate analysis of factors associated with clinically relevant antimicrobial omission frequency.

Characteristic	Number of clinically relevant omissions	Number of doses scheduled	Omission per 100 doses scheduled	p-value
**Dose Schedule**				
Once per week	0	37	0.00	p<0.001
Twice per week	0	17	0.00	
Every 3 days	3	40	7.50	
3 times per week	180	1710	10.53	
Every 2 days	5	392	1.28	
4 times per week	2	188	1.06	
Every 36 hours	1	80	1.25	
5 times per week	1	55	1.82	
6 times per week	0	4	0.00	
Every 24 hours or once daily	574	45753	1.25	
Every 18 hours	2	192	1.04	
Every 12 hours or twice daily	1596	83983	1.90	
Every 10 hours	0	2	0.00	
Every 8 hours or 3 times daily	1047	70267	1.49	
Every 6 hours or four times daily	1368	77857	1.76	
5 times daily	35	657	5.33	
Every 4 hours	89	8101	1.10	
7 times per day	0	3	0.00	
**Medication Route**				
Solid oral	2951	140755	2.10	p<0.001
Liquid oral	402	14426	2.79	
Parenteral	1512	133708	1.13	
Inhaled	38	449	8.46	
**Shift (dose scheduled)**				
Day (0700-1459H)	2462	124193	1.98	p<0.001
Evening (1500-2259H)	1805	118911	1.52	
Night (2300-0659H)	636	46234	1.38	
**Hospital**				
**1**	2400	125145	1.92	p<0.001
**2**	1206	82280	1.47	
**3**	1297	81913	1.58	

**Table 3:** Results: Medication Administration Omissions by Reason Category and Clinical Relevance.

Reason description	Total (%)^1^	Not relevant (%)^1^	Clinically Relevant (%)^1^	% Relevant
Technical issue	3552 (35.55)	3525 (65.54)	27 (0.55)	0.76%
No IV access	396 (3.85)	54 (1.00)	342 (6.98)	86.36%
No GI access	422 (4.10)	48 (0.89)	374 (7.63)	88.63%
Patient unavailable	389(3.78)	103 (1.91)	286 (5.83)	73.52%
Other or not noted	1894(18.42)	157 (2.92)	1737 (35.43)	91.71%
Medication not available	609(5.92)	278 (5.17)	331 (6.75)	54.35%
Patient condition	259(2.52)	27 (0.50)	232 (4.73)	89.58%
Patient preference	1579(15.36)	124 (2.30)	1455 (29.68)	92.15%
ADR to drug	44(0.43)	4 (0.07)	40 (0.82)	90.91%
Scheduling issue	850(8.27)	779 (14.48)	71 (1.45)	8.35%
Serum drug concentration high	266(2.59)	266 (4.94)	0(0.00)	0.00%
Awaiting serum drug concentration	22(0.21)	14 (0.26)	8 (0.16)	36.36%
Total	10282(100)	5379 (100)	4903 (100)	47.65%

^1^ Percentage refers to fraction of column total

In the logistic regression model, we transformed dosing frequency from an ordinal variable to a binary variable for irregular (<1 time per day, five times daily, q18H, etc.) or regular frequency (1,2,3,4 or 6 times per day) because of the obvious non-linearity of association of frequency of dosing with omission rate in bivariate analysis (Table 2). We identified route and nursing shift as significant factors associated with clinically relevant MAO (Table 4). Since the hospital site variable is specific to our regional healthcare organization, we repeated the analysis without this factor and found the model to be consistent with the first in terms of the same other significant factors, odds ratios and 95% confidence intervals (Table 4).

**Table 4:** Multivariate Logistic Regression Models of Clinically Relevant MAO.

Model 1—Hospital Included				
Factor	OR	95% Confidence Interval		p
Hospital 1	1.23	1.15	1.32	<0.0001
Hospital 2	0.88	0.81	0.96	0.02
Hospital 3	reference			
Irregular frequency	3.55	3.09	4.08	<0.0001
Inhaled route	5.24	3.73	7.30	<0.0001
Liquid oral	1.41	1.26	1.56	<0.0001
Parenteral	0.53	0.50	0.57	<0.0001
Solid oral	reference			
Day (07:01–1500)	0.98	0.89	1.08	0.73
Evening (15:01–23:00)	0.78	0.71	0.86	<0.0001
Night (23:01–07:00)	reference			
**Model 2—Hospital excluded**				
Irregular frequency	3.546099	3.08642	4.081632653	<0.0001
Inhaled route	4.59	3.28	6.41	<0.0001
Liquid oral	1.40	1.26	1.55	<0.0001
Parenteral	0.54	0.51	0.58	<0.0001
Solid oral	reference			
Day (07:01–1500)	0.98	0.89	1.08	0.65
Evening (15:01–23:00)	0.78	0.71	0.86	<0.0001
Night (23:01–07:00)	reference			

Overall, the most common reason for a missed dose was a technical issue (Table 3). For most of these MAO, it was usually because the order was intended to be stopped but the care team had not actually discontinued the order in the computer system. These omissions were considered not clinically relevant. However, amongst omissions regarded as clinically relevant, a lack of explanation was most common, followed by “patient preference or refusal,” “no GI access,” “no IV access”, “medication not available”, “patient unavailable”, “patient condition”, “scheduling issue” and “adverse drug reaction to drug” in frequency of relevant dose omissions (Table 2). The twenty most frequent omissions with respect to drug products (generic name and dosage form) are listed in Table 5 along with the 5 most common reasons for omissions.

**Table 5:** Top 20 drugs for medication administration omissions and documented reason for omission.

Drug Product	Relevant Omission	Performed	Total	Relevant omit/100 doses of this drug	Relevant Omission Reason Code (5 most frequent in order of most to least frequent, refer to Table 1 for explanation of codes)					Top 5 reasons account for
					1	2	3	4	5	
metronidazole tab	486	22487	22973	2.1	8	5	3	7	4	94.65%
ciprofloxacin tab	469	22098	22567	2.1	8	5	3	6	7	93.18%
piperacillin / tazobactam inj	466	42582	43048	1.1	5	2	8	6	4	95.28%
sulfamethoxazole / trimethoprim DS tab	345	7307	7652	4.5	5	8	3	6	7	94.49%
cephalexin tab	277	13928	14205	2.0	8	5	6	4	3	93.14%
nitrofurantoin tab	274	8818	9092	3.0	8	5	4	6	3	88.64%
vancomycin inj	174	9776	9950	1.7	5	2	8	4	6	96.00%
vancomycin liquid	165	8399	8564	1.9	5	8	6	3	7	90.30%
cefazolin inj	164	15576	15740	1.0	5	2	4	6	8	97.56%
amoxicillin / clavulanate tab	163	9213	9376	1.7	5	8	3	7	6	92.02%
metronidazole inj	150	12387	12681	1.2	5	2	8	4	6	94.33%
levofloxacin tab	146	12297	12443	1.2	8	5	3	4	6	93.15%
amoxicillin cap	140	6315	6455	2.2	8	5	3	6	4	92.91%
ceftriaxone inj	118	14063	14181	0.8	5	2	8	4	6	95.73%
clindamycin cap	79	2136	2215	3.6	8	5	7	3	6	94.94%
acyclovir tab	66	3922	3988	1.7	5	8	4	3	6	98.48%
cefuroxime tab	64	3014	3078	2.1	8	5	3	7	6	98.44%
azithromycin tab	59	3822	3881	1.5	5	8	3	7	6	94.92%
clarithromycin tab	57	2966	3023	1.9	8	5	3	7	4	94.83%
meropenem inj	56	5653	5709	1.0	5	2	8	4	6	98.25%

## Discussion

We performed a large data set analysis of MAO of antimicrobials on inpatient medical wards to determine the frequency of MAO, describe reasons for MAO and identify predictive factors for MAO. Our focus was on clinically relevant MAO but many of the non-relevant omissions were due to reasons such as technical errors or rescheduling of doses (Tables 1 and 3), which would be unlikely to appear as an omission in a paper-based MAR record. This demonstrates the need for a flexible system permitting rescheduling and the complexity of the medication administration process for inpatients. Of the relevant omissions unspecified reason (no explanation provided by nurse, 35.2%), patient preference (29.8%) and access issues (for vascular or gastrointestinal access, 14.7%) were the most common reasons for omissions documented by nursing staff, and these reasons were relatively consistent across different drug products. When no explanation was provided we classified this as clinically relevant to determine the most conservative estimate of relevant dose omission rate.

Infrequent and unusual dosing schedule (every 2 days, every 5 hours, every 18 hours, etc.) was associated with a higher relevant omission rate even though the scheduling is automatized by the computer system. A possible explanation for this is that many prophylactic antimicrobials are scheduled on infrequent and irregular schedules and may be more easily missed.

It was noted that the oral route (solid or liquid) oral was associated with higher rates of omissions compared to intravenous by bivariate (2.10, 2.79 relevant omissions per 100 scheduled doses vs 1.13 respectively [p<0.001, chi-squared]) and adjusted analyses (OR 1.41, 95% CI 1.26–1.56 and OR 0.53, 95% CI 0.50–0.57 for liquid oral and parenteral, respectively, compared to the reference solid oral) analyses.

We may only speculate as to why oral medications were associated with a higher rate of MAO than parenterally administered antimicrobials. It may be that oral medications are seen as less important or that patient conditions (eg. Loss of consciousness) are more likely to impact the administration by oral route. Since antimicrobial stewardship programs encourage the early adoption of orally administered antimicrobial therapy in the course of a treatment regimen, health care providers must ensure that patients will have an equal chance of receiving all doses, regardless of route.

We also noted that nursing shift was associated with MAO. In adjusted analysis, the evening shift was associated with a significantly lower rate of MAO (OR 0.79, 95% CI 0.71–0.87) although in crude analysis the night shift had a slightly lower rate (1.38 vs 1.52 MAO/100 scheduled doses, p = 0.031) which may not be clinically relevant. As many patient activities and events are concentrated during day shift times, the higher rate (1.98 MAO/100 scheduled doses) observed may reflect more difficulty in getting patient, nursing staff and drug together during this shift.

We noted that one site had a statistically higher rate of MAO of antimicrobials which may indicate better medication management at the site with the lower rates or possibly the higher patient acuity at site 1 which is a tertiary acute care centre.

A recent, large sample study from a public hospital in the UK using similar data to ours found 12.4% of doses of all medications were not administered.[14] The investigators also developed a *de novo* classification scheme using an Ishikawa (fish-bone) diagram which mapped themes and domains of dose omission reasons. The authors reported the proportions of each reason for admission in terms to total missed doses. Their framework consisted of 8 domains and 54 specific themes for omissions. The most common medication being acetaminophen and the most common reason was “patient refused” which was the reason given in 45.0%, which is much higher than our observed results examining only antimicrobials of 15.36% (Table 3, “Patient Preference”).[14] Our study adds to these findings in that we reported omissions by additional factors such as route, frequency of administration and time of day (nursing shift). Another study by the same investigators using a similar data source found antimicrobial omission rates of 4.4% to 10.3% of prescribed doses in post and pre intervention periods, respectively.[15]

Aside from these latter studies and our current study, data regarding dose omissions events have usually only been retrievable from medical error observation studies or paper chart review studies.^1-3,9-11^ Dose omission frequency in observation and paper chart studies was reported to range from 0.58% to 12.60%. The studies performed in critical care settings[9,11] tended to have omission frequencies on the low end of this range and differ from our study in that all types of medication were included. In our study, we found a relatively low rate of MAO on medicine wards (3.49% and 1.66% of all and clinically relevant MAO, respectively), but we restricted our investigation to antimicrobial medications.

As many relevant omissions occur for reasons that could not be attributed to an error on part of a health care worker, eg. No IV access, no GI access, patient not on ward, it may be inappropriate to label omissions as errors. The term “adherence” in the outpatient setting has largely replaced the term “compliance”, in recognition that patients are not necessarily wholly responsible for following the plans devised by health care professionals that may not fit individual patient’s circumstances.[16,17] In parallel, within the hospital system, medication administration requires the alignment and performance of several events by prescribers, pharmacy and hospital transportation systems, nursing staff and a willing and able patient. Therefore it should be recognized that in medically complex inpatient settings, treatment and assessment procedures may result in multiple conflicts whereby it may impractical or impossible for 100% adherence to the drug therapy plan. To address this, we propose the term “inpatient adherence” be used. We believe inpatient adherence is a quality and safety issue within the inpatient setting which is relevant for further investigation and perhaps periodic surveillance.

We acknowledge the limitations of our study: Firstly, we relied on the real-life records of nurses documenting administered doses of antimicrobials without independent verification and as such there may be random or systemic discrepancy between what was entered and what the patient actually received. This limitation is balanced against the limitations of typical studies looking at this subject, for instance the observer effect and the labour and/or lower sample size. Secondly, there were hundreds of differing short explanatory notes (maximum 255 characters) entered with “not performed” doses, most of which were easily interpretable but others were possible to interpret in different ways. However, a second, independent assessment of a sample of dosing records demonstrated good agreement with the primary assessor (k = 0.68 for category of omission and k = 0.75 for relevance), so we believe our assessment procedure to be reliable. In addition, although we assume that medication dose omissions, especially antimicrobials, may result in patient harm but assessment of this was beyond the scope of our inquiry so we do not know if this indeed was the case. Finally, our findings may be very specific to our patient care management system and/or our nursing practices, however, the subject of our study may be of interest to the fields of nursing, pharmacy and patient safety and our novel assessment method, may be applicable elsewhere.

We believe clinically MAO, especially concerning antimicrobials in acute care patients, to be an important health care quality issue, deserving of further research in the future. Investigations examining other predictors of clinically relevant MAO such as patients age, race, gender, admitting diagnosis, medical comorbidities and acuity or health system variables such as hospital pharmacy dispensing systems, nursing workload or nurse to patient ratios may improve the general understanding of this topic.

In conclusion, we performed a large cohort study of acute care medicine ward patients and found MAO of antimicrobials to be relatively uncommon, but not rare incidents and furthermore, approximately half of dose omissions were potentially clinically relevant. Our data indicate that oral route was associated with more dose omissions compared to intravenously administered antibiotics.
